# Dying cells expose a nuclear antigen cross-reacting with anti-PD-1 monoclonal antibodies

**DOI:** 10.1038/s41598-018-27125-6

**Published:** 2018-06-11

**Authors:** Philipp Metzger, Sabrina V. Kirchleitner, Lars M. Koenig, Christine Hörth, Sebastian Kobold, Stefan Endres, Max Schnurr, Peter Duewell

**Affiliations:** Center of Integrated Protein Science Munich (CIPSM) and Division of Clinical Pharmacology, Medizinische Klinik und Poliklinik IV, Klinikum der Universität München, Munich, Germany

## Abstract

Checkpoint molecules such as programmed death 1 (PD-1) dampen excessive T cell activation to preserve immune homeostasis. PD-1-specific monoclonal antibodies have revolutionized cancer therapy, as they reverse tumour-induced T cell exhaustion and restore CTL activity. Based on this success, deciphering underlying mechanisms of PD-1-mediated immune functions has become an important field of immunological research. Initially described for T cells, there is emerging evidence of unconventional PD-1 expression by myeloid as well as tumor cells, yet, with cell-intrinsic functions in various animal tumor models. Here, we describe positive PD-1 antibody staining of various murine immune and tumour cells that is, unlike for T cells, not the PD-1 receptor and restricted to cells with low forward scatter characteristics. Based on flow cytometry and various approaches, including two established murine anti-PD-1 antibody clones, CRISPR/Cas9 genome editing and confocal imaging, we describe a staining pattern assigned to a nuclear antigen cross-reacting with anti-PD-1 monoclonal antibodies. Lack of PD-1 expression was further underlined by the analysis of PD-1 expression from B16-F10-derived 3D cultures and *ex vivo* tumours. Thus, our data provide multiple lines of evidence that PD-1 expression by non-T cells is unlikely to be the case and, taking recent data of PD-1 tumour cell-intrinsic functions into account, suggest that other antibody-mediated pathways might apply.

## Introduction

The quality of innate and adaptive immune cell activation pathways underlies a sensitive balance that is, at least in parts, regulated by immune checkpoints to maintain immune homeostasis^1^. Checkpoint blockade has substantially improved the therapy of several cancer types including melanoma^2^, non-small cell lung cancer^3,4^ as well as head and neck squamous cell carcinoma^5^, and holds promise for a variety of mismatch repair-deficient tumours, for example those found in colorectal cancer^6^. Within immune checkpoints discovered today, programmed cell death 1 (PD-1) is one of the best-characterized molecules and the therapeutic application is based on the role of PD-1 in regulation of T cell function, as it alters metabolic and cell cycle processes^7^. Under physiological conditions, PD-1 dampens immune responses by inhibiting T cell activation, otherwise leading to immune-mediated pathologies^8^.

The redundancy of inhibitory pathways is also hijacked by tumours to cause T cell exhaustion, which then results in tumour immune evasion. While the ligand for PD-1 receptor, PD-L1, is expressed on various immune and non-immune cells including tumour cells, PD-1 receptor expression and function have recently been shown not only for T cells, but also for B cells and other cells of the innate immune system^9–12^. Even more surprising, a recent report described PD-1 expression in a subset of murine melanoma cells, which promoted tumour growth in a cell-intrinsic manner. This non-canonical concept, however, clearly challenges the cancer immunology field to revisit the general concept of anti-PD-1-directed therapies, initially assumed to exclusively target T cells in tumour bearing hosts^13^.

Unexpected PD-1 expression on cells other than T cells is quite intriguing and greatly enhances the field of immunological research, with potential implications in tumor therapy. Hence, recent advances in this field warrant further clarification and prompted us to investigate PD-1 expression on several murine immune and non-immune cells, including various tumour models. However, there is a thin line between carefully controlled experimental procedures and data interpretation, where recent study designs rather fell short. A major hurdle involved in the experimental design ist the choice of validated and reliable key resources of tools that allow retrospective data analysis and conclusions. Thus, poor reproducibility of published results is still a critical issue, which is mostly based on a insufficiently-described methodology or questionable antibodies. Antibodies are the backbone of protein science, however, earlier studies have revealed that less than 50% actually suffuciently meet desired quality requirements^14^. With this is mind, we aimed at validating two widely-used murine anti-PD-1 antibody clones, 29 F.1A12 and RMP1-14, which are known to target PD-1 and block binding to its ligand PD-L1. Based on flow cytometry, we compared PD-1 expression of various immune and non-immune cells to the canonical PD-1 expression profile of T cells. By employing tightly controlled FACS- and image-based validation approaches in wild-type and PD-1-deficient cells, we identified a cross-reactive nuclear antigen that becomes available in dead or dying cells. In summary, we confirmed PD-1 staining of T cells for both antibody clones used; however, applying well-controlled gating strategies, tumour cells and other immune cellswere found negative for PD-1 expression, thus, challenging interpretation of recently published animal models.

## Results and Discussion

### Expression of PD-1 by immune cells populations in spleens of tumour-bearing mice

**Figure 1:**
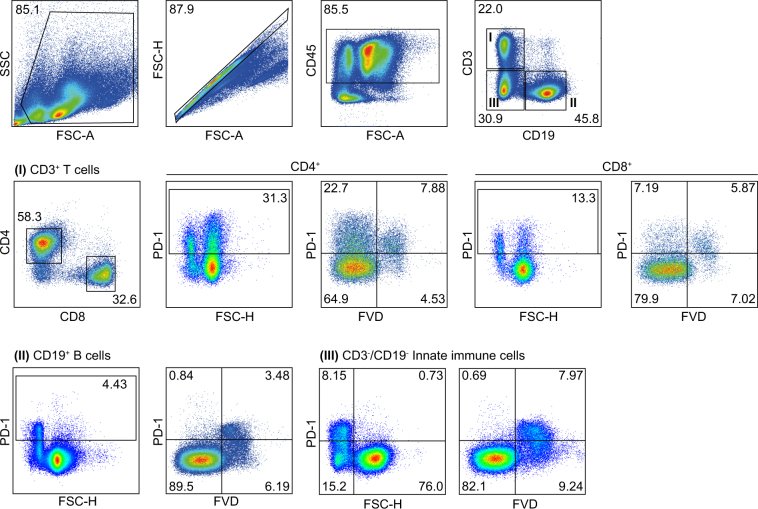
Expression of PD-1 by immune cell populations in spleens of tumour-bearing mice. Spleens of mice with orthotopic pancreatic tumours were removed to assess immune cell populations by flow cytometry. Live cell (FCS-A/SSC) and singlet (FSC-A/FSC-H) cell gating was done. Anti-CD45 antibody was applied for assessing immune cell populations, which were subdivided into CD3^+^CD4^+^ as well as CD3^+^CD8^+^ T cells (gate I), CD19^+^ B cells (gate II) and CD3^-^CD19^-^ innate immune cells (gate III). PD-1 (clone 29F.1A12) and fixable viability dye (FVD) staining was assessed by flow cytometry. Gates I – III show PD-1/FSC-H and PD-1/fixable viability dye (FVD) staining of respective cell populations. One out of three independent experiments is shown.

Amongst the plethora of suppressive mechanisms, the PD-1/PD-L1 axis represents one of the most potent inhibitory signalling cascades to abort T cell-mediated tumour killing. Tumour-derived factors lead to an upregulation of PD-1 expression in tumour-infiltrating T cells and potentially other immune cell types, such as B cells and innate immune cells^9–11,15,16^. To study PD-1 expression by immune cell subsets in tumour bearing hosts, mice were challenged with a GEMM-derived orthotopic pancreatic tumour^17^ and splenic immune cell populations were assessed by flow cytometry. The gating strategy included live cell and singlet gates, combined with CD45 to identify immune cells. We added FcRII/FcRIII blocking antibodies prior staining for CD3, CD4, CD8 and CD19 to investigate T and B cells (I and II). In addition, we added a third gate (III) to include remaining cells of the innate immune system (Fig. 1). PD-1 was expressed by CD4^+^ and CD8^+^ T cells (Fig. 1, gate I). In addition, PD-1 staining (clone 29F1A12) uncovered a FSC-H^low^ population predominantly positive for PD-1. As we routinely included the fixable viability dye (FVD) for dead cell exclusion, we observed that the FSC-H^low^ PD-1^+^ population mainly contained dead cells. In addition, PD-1 expression has been also shown for B cells as well as other innate immune cells under malignant, infectious or autoinflammatory conditions^9–11,15,16^. Following the same gating strategy, we observed a similar FSC-H^low^ staining pattern in B cells and innate immune cells; however, FVD staining confirmed PD-1 staining only for dead cells (Fig. 1, gate II and III). Based on this data, PD-1 expression was limited to T cells in this tumour model.

### PD-1-specific mAb recognize an antigen exposed by dead cells

**Figure 2:**
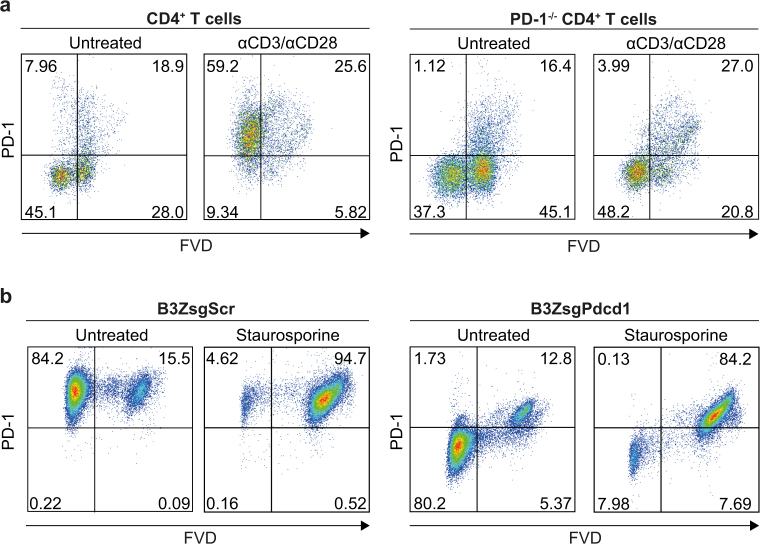
PD-1-specific mAb recognize an antigen exposed by dead cells. (**a**) Wild-type or *Pdcd1*^*−/−*^ mice were stimulated with anti-CD3/anti-CD28 beads for 48 h or left untreated. Anti-PD-1 (clone 29 F.1A12) and fixable viability dye (FVD) staining of CD4^+^ T cells was assessed by flow cytometry. (**b**) The B3Z hybridoma T cell line was targeted with CRISPR/Cas9, by using a scrambled (B3ZsgScr) or a PD-1-directed (B3ZsgPdcd1) sgRNA, and treated with staurosporine (1 µM) or left untreated. PD-1 as well as FVD staining was studied by flow cytometry. One out of three independent experiments is shown.

A decrease in the forward scatter (FSC-H^low^) of PD-1^+^ populations indicates alterations in cellular volume, as observed in dying cells. PD-1 staining (clone 29 F.1A12) in these cells could be due to binding of an intracellular PD-1 pool that becomes accessible in cells with leaky membranes. On the other hand, off-target binding of the antibody can either occur via ionic or hydrophobic interactions or stickiness of dying cells that release high amounts of DNA^18,19^. To address this question, we analysed PD-1 expression in primary CD4^+^ T cells from wild-type and *Pdcd1*^*−/−*^ mice. As expected, wild-type CD4^+^ T cells, but not PD-1^*−/−*^ T cells, expressed PD-1. This observation became even more pronounced after T cell activation with anti-CD3/anti-CD28 beads, clearly demonstrating the specificity of the anti-PD-1 mAb within viable (FVD^−^) cells (Fig. 2a). Interestingly, we detected PD-1/FVD double-positive cells in wild-type as well as in PD-1^*−/−*^ cells. Thus, PD-1 staining in the dead cell fraction is due to cross-reactivity. To confirm these data in a second model, we performed CRISPR/Cas9-targeted PD-1 deletion in the B3Z hybridoma T cell line. B3Z T cells (B3Z-sgScr) intrinsically express high PD-1 levels, which was absent in PD-1-deleted (B3Z-sgPdcd1) cells (Fig. 2b). Again, PD-1 mAb staining (clone 29 F.1A12) was found in both PD-1 wild-type and knock-out cells in the dead cell fraction, indicative of unspecific binding. This became even more evident when cells were treated with the apoptosis inducer staurosporine (Fig. 2b). Our data clearly underline the necessity of a strict gating strategy to rule out false positive findings induced by dying or dead cells.

### B16-F10 melanoma cells do not express PD-1

**Figure 3:**
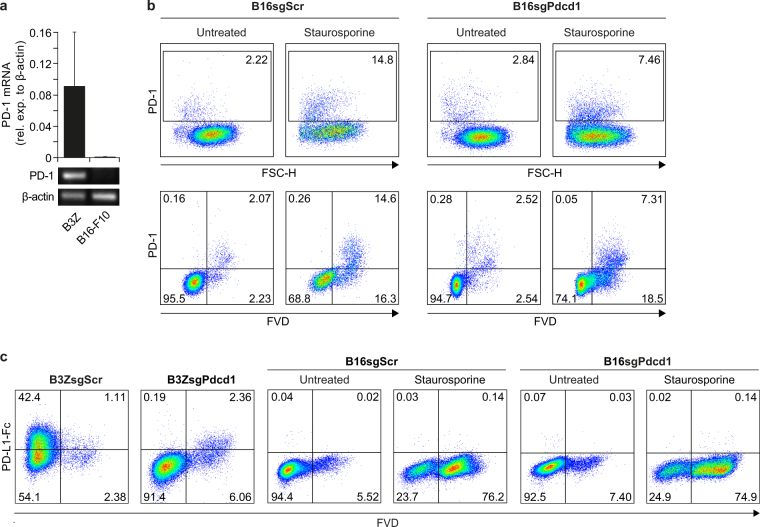
B16-F10 melanoma cells do not express PD-1. (**a**) PD-1 mRNA expression of B3Z and B16-F10 melanoma cells was assessed by qRT-PCR. Cropped blots show qRT-PCR transcripts using 3% agarose gel electrophoresis (for full blot, see Supplementary Figure S4). (**b**) CRISPR/Cas9-targeted B16-F10 control (B16sgScr) and PD-1^*−/−*^ (B16sgPdcd1) cells were cultured in the absence or presence of staurosporine (1 µM). Anti-PD-1 antibody (clone 29 F.1A12) and fixable viability dye (FVD) staining was analysed by flow cytometry. (**c**) B3Z control (B3ZsgScr) and PD-1^*−/−*^ (B3ZsgPdcd1) as well as B16-F10 control (B16sgScr) and PD-1^*−/−*^ (B16sgPdcd1) cells were treated with staurosporine (B16-F10) or left untreated (B3Z and B16-F10). Cells were stained with recombinant mouse PD-L1-Fc fusion protein and FVD. One out of three independent experiments is shown.

A recent publication attracted our attention showing B16 melanoma-intrinsic PD-1 receptor functions supporting tumour growth^13^. The authors claim dead cell exclusion; however, the PD-1^+^ subpopulation of B16-F10 melanoma cells shown in this work exhibits FSC-H^low^ properties, as compared to the main PD-1^−^ cell population. Thus, we were interested in PD-1 expression of B16-F10 cells and first assessed mRNA expression levels by RT-PCR. B3Z cells served as positive control, expressing high PD-1 mRNA levels. In contrast, we could not detect PD-1 mRNA in B16-F10 cells. To confirm our data and to rule out sensitivity limitations, we additionally performed CRISPR/Cas9-targeted gene knockout in B16-F10 melanoma cells. PD-1 knockout single cell clones were validated by Sanger sequencing of individual alleles (Supplementary Fig. S1a). We assessed PD-1 expression by flow cytometry and compared the two mainly used antibody clones (29 F.1A12 and RMP1-14), which were also included in the above-mentioned study. We found a fraction of approximately 2–5% PD-1^+^ tumour cells with FSC-H^low^ properties, quite similar to published results^13^ (Fig. 3b). This staining pattern was seen in control and PD-1 knock-out tumour cells, indicative of unspecific binding. To demonstrate that the FSC-H^low^ population contained dead cells, we co-stained cells with fixable viability dye (FVD). We observed PD-1 staining exclusively in the dead cell fraction for both clones 29 F.1A12 and RMP1-14 mAb, respectively. Again, this staining pattern became more evident by staurosporine treatment of the tumour cells (Fig. 3b and Supplementary Fig. S1b). The results were also confirmed by other cell death markers, such as 7-AAD and propidium iodide (Supplementary Fig. S1c). Of note, PD-1 staining of dead cells was not limited to the B16-F10 melanoma cell line, but also observed for other tumour entities, including hepatocellular carcinoma and pancreatic cancer cell lines (Supplementary Fig. S2).

Next, we investigated binding of PD-1 to its natural ligand by using recombinant Fc-tagged PD-L1. Binding was only observed in the B3Z wild-type cell line, but not in PD-1-deficient B3Z or wild-type B16-F10 tumour cells, providing further evidence for the lack of PD-1 expression in B16-F10 cells (Fig. 3c). It is conceivable that variations in cell culture conditions lead to altered gene expression, an exciting approach in cancer stem cell (CSC) research^13,20^, which has been applied for B16 melanoma cells by using different geometric culture shapes^21^. Here, the authors found the CSC-related marker ABCB5 and correlated it to the expression of costimulatory B7.2 and PD-1 in established melanoma^22^. We made use of an *in vitro* ‘hanging drop’ culture system to grow B16-F10 cells as three-dimensional spheres (Supplementary Fig. S3a). Furthermore, we analysed PD-1 expression of freshly-isolated B16-F10 tumour cells (Supplementary Fig. S3b). Again, we were unable to detect PD-1 expression in viable tumour cells for all approaches tested, including two- and three-dimensional culture methods as well as *in vivo* conditions.

### Anti-PD-1 mAb is cross-reactive with a nuclear antigen

**Figure 4:**
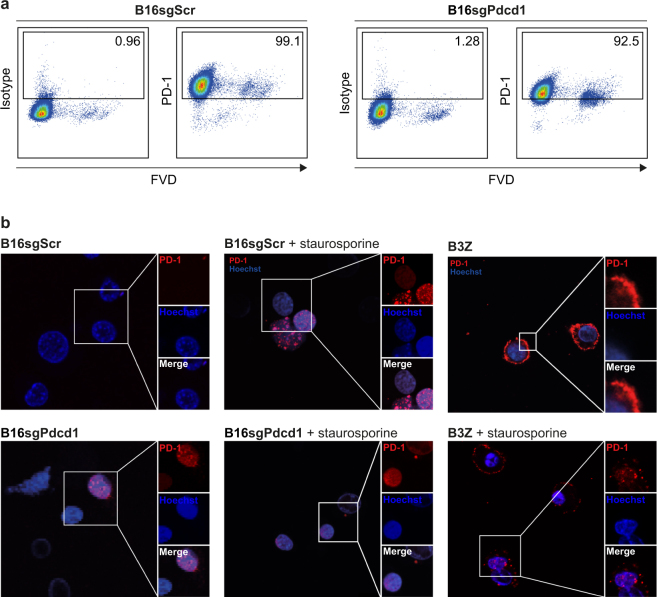
PD-1 mAb is cross-reactive with a nuclear antigen. (**a**) CRISPR/Cas9-targeted B16-F10 control (B16sgScr) and PD-1^*−/−*^ (B16sgPdcd1) cells were stained with FVD, permeabilized, stained with anti-PD-1 antibody (clone 29 F.1A12) and analysed by flow cytometry. (**b**) B16-F10 control (B16sgScr) cells as well as B3Z cells were treated with staurosporine or left untreated prior to anti-PD-1 antibody staining (clone 29 F.1A12) (red). Nuclei were counterstained with Hoechst 33342 (blue). Localization of anti-PD-1 staining pattern was analysed by confocal microscopy. Representative data of two independent experiments are shown.

Isotype-controlled PD-1 staining of FVD^+^ cells suggests cross-reactivity of anti-PD-1 mAb with an antigen accessible in dead cells. Indeed, intracellular staining (clone 29 F.1A12) of both wild-type and PD-1^*−/−*^ B16-F10 cells revealed staining in nearly all B16-F10 control (B16sgScr) and PD-1^*−/−*^ (B16sgPdcd1) melanoma cells, as assessed by flow cytometry (Fig. 4a). To address the question of the subcellular localization, we performed confocal microscopy comparing B3Z cells with untreated and staurosporine-treated B16-F10 melanoma cells. As expected, untreated B3Z cells showed strong plasma membrane-associated PD-1 staining (clone 29 F.1A12), which was absent in B16-F10 cells. Staurosporine treatment unravelled a significant proportion of B16-F10 cells that stained positive with the anti-PD-1 antibody. This was independent of PD-1-expression and was strictly located within the nucleus (Fig. 4b). Of note, sparse cells within untreated PD-1^*−/−*^ B16-F10 cells also showed the nuclear staining pattern, indicative of spontaneous cell death with antibody passage via leaky membranes.

From our experiments we conclude that B16-F10 melanoma cells and other tumour cell lines do not express PD-1. In fact, PD-1 staining of tumour cells is due to cross-reactivity of anti-PD-1 mAb with a nuclear antigen that only becomes accessible in dying cells, possibly through leaky membranes. Here, our data clearly shows that the concept of PD-1 expression on tumour cells, including cell-intrinsic downstream effects, has to be challenged. In addition, the comprehensive approach of validated antibody sources should be enhanced by careful gating strategies, which is accompanied by dead cell exclusion, and routinely used when studying PD-1 expression in T cells and other cell types. Antibodies are the supporting pillar in biological sciences and pivotal to pave the road from bench to bed-side, starting development in animal models. At the point where we tested two major murine antibody clones for their PD-1 specificity it is important to state that another recently used goat anti-mouse PD-1 antibody was already discontinued, thus, evading our validation efforts. Thus, further investigations are necessary to revisit the basic immunological concept with possible antibody-mediated implications, when working with anti-PD-1 antibodies in animal models.

## Material and Methods

### Mice, tumour induction and cell isolation

C57BL/6 J mice were purchased from Janvier Labs (Le Genest-Saint-Isle, France) and Pdcd1 knockout mice were a kind gift from Prof. Protzer (Institute of Virology, Technical University of Munich, Germany). Experimental animal procedures conformed to the guidelines and regulations of the Animal Care Committee and were approved by the local government (Regierung von Oberbayern, Maximilianstrasse 39, 80538 Munich; GZ 55.2-1-54-2532-175-2012). For tumour induction, 2 × 10^5^ T110299 cells were orthotopically implanted into the pancreas of C57BL/6 J mice. After 21 days, mice were sacrificed and spleens were removed for cell isolation. Spleens were meshed and passed through a 70 µm cell strainer (Miltenyi SmartStrainer, Miltenyi, Bergisch Gladbach, Germany) to obtain a single cell suspension. Red blood cell lysis (BD Pharm Lyse™, BD Biosciences, Heidelberg, Germany) was done prior antibody staining for flow cytometric analysis. For the B16-F10 tumour model, 1 × 10^5^ cells were injected s.c. into the flanks of C57BL/6 mice. 19 days after engraftment, tumours were removed and processed into single cell suspensions using collagenase and DNase (Sigma-Aldrich, Munich, Germany) at 37 °C for 30 minutes. Tumour cells were filtered through a 100 µm mesh, washed and stained for flow cytometer analysis.

### Cell culture and reagents

B16-F10 (ATCC; RRID:CVCL_0159), Hep-55.1 C (CLS; RRID: CVCL_5766), Hepa1-6 (ATCC; RRID: CVCL_0327), RIL175 (Prof. Greten), Pan02 (Prof. Lauber) and T110299 (Prof. Siveke; Ptf1a-Cre LSL-K_ras_^G12D^ LSL-Trp53^fl/R172H^) cells were cultured in DMEM (Lonza, Cologne, Germany) supplemented with 10% fetal calf serum (FCS; ThermoFisher Scientific, Darmstadt, Germany), 2 mM L-glutamine (Lonza, Cologne, Germany) and 10 µg/ml ciprofloxacin (Fresenius Kabi, Bad Homburg, Germany). B3Z T cell hybridoma cells were cultured in RPMI 1640 (Lonza, Cologne, Germany) supplemented with 10% FCS, 2 mM L-glutamine and 10 µg/ml ciprofloxacin. Splenocytes were cultured in RPMI 1640 supplemented with 10% FCS, 2 mM L-glutamine, 100 U/L penicillin, 0.1 mg/ml streptomycin (all Lonza, Cologne, Germany), 1 mM sodium pyruvate (Biochrom, Berlin, Germany) and 0.1 mM non-essential amino acids (ThermoFisher, Darmstadt, Germany). For T cell activation, cells were seeded at 1 × 10^6^ cells/0.5 ml in a 48-well plate, in the presence or absence of anti-CD3/anti-CD28 Dynabeads^®^ (ThermoFisher, Darmstadt, Germany). Cells remained in culture for 48 h and were subsequently processed for flow cytometry. For cell death induction, cells were treated with 1 µM staurosporine (Sigma Aldrich, Munich, Germany) for 4–6 hours.

### Generation of PD-1 knockout cell lines

PD-1 knockout cells were generated using a lentiviral CRISPR/Cas9 method as described^23,24^. In brief, sgRNA sequences were obtained from GeCKOv2 mouse library. DNA oligonucleotides encoding for the sgRNA sequences and a BsmBI restriction overhang were cloned into a pLentiCRISPRv2_ccdB_Cas9-P2A-Puro vector (a gift from Marc Schmidt-Supprian, TU Munich). HEK 293 T cell were transfected with the sgRNA encoding pLentiCRISPRv2 vector, pcMV_dR8-74 and pVSVG (Addgene). After 48 hours, lentivirus-containing supernatant was transferred to target cells (B16-F10 and B3Z). After further 48 hours, transduced cells were treated with puromycin for positive selection. PD-1 negative B3Z cells were single-cell sorted into a 96-well plate using BD FACSAria^TM^ III cell sorter (BD Biosciences, Heidelberg, Germany). Single-cell clones of B16-F10 cells were obtained by serial dilution. For verification of PD-1 knockout, genomic DNA was isolated using the QuickExtract™ DNA Extraction Kit (Epicentre, Madison, WI, USA). After target amplification, genomic editing was verified by T7 endonuclease digest as described^25^. For B16F10 cells, individual alleles were cloned into a plasmid and sequenced by Sanger sequencing (Eurofins Genomics, Ebersberg, Germany).

### FACS analysis

Single cell suspensions were incubated with anti-PD-1 mAb (1:200 dilution; clones 29 F.1A12 or RMP1-14; both BioLegend, London, UK) in PBS containing FCS (1%) and EDTA (2 mM) (staining buffer) for 30 min on ice. Dead cell staining was included as indicated, using eFluor 780-conjugated fixable viability (FVD; 1:5000; ThermoFisher, Darmstadt, Germany), propidium iodide (1 µg/ml; Sigma-Aldrich, Munich, Germany) or 7-AAD (2.5 µg/ml; ThermoFisher, Darmstadt, Germany). Intracellular staining was done using the Foxp3/Transcription Factor Staining Buffer Set according to the manufactures One-step protocol (ThermoFisher, Darmstadt, Germany). In brief, following dead cell staining with the FVD, cells were fixed, permeabilized and subsequently stained with the anti-PD-1 antibody. Samples were analysed with the BD FACSCanto^TM^ II and BD LSRFortessa^TM^ flow cytometer (BD Biosciences, Heidelberg, Germany).

### qRT-PCR

B3Z and B16-F10 total RNA was isolated with the peqGOLD Total RNA Kit (VWR International, Darmstadt, Germany) and adjusted RNA was transcribed into cDNA using the RevertAid First Strand cDNA Synthesis Kit (ThermoFisher, Darmstadt, Germany), according to the manufacturers’ protocol. qRT-PCR was done with the Kapa Probes Fast Universal Kit (Kapa Biosystems, Roche, Penzberg, Germany) on a LightCycler® 480 II instrument (Roche, Penzberg, Germany), using the Roche Universal Probes library for the primer design of β-actin and PD-1. For visualization of mRNA transcripts, qRT-PCR samples were loaded onto a 3% agarose gel (Tris-acetate-EDTA buffer) and stained with SERVA DNA Stain Clear G (Serva Electrophoresis GmbH, Heidelberg, Germany). Images were acquired using the AlphaImager^®^ with the AlphaEase^®^ FC Software v6 (Alpha Innotech, Kasendorf, Germany).

### ‘Hanging drop’ 3D culture

1 × 10^2^ B16-F10 cells were seeded on the lid of a non-adherent plate in 50 µl culture medium. After seeding, the lid was turned upside down. Cells were cultured for up to 10 days in a humidified incubator at 37 °C with 5% CO_2_.

### Confocal microscopy

Glass slides (ThermoFisher, Darmstadt, Germany) were treated overnight with 1 M HCl, washed with PBS and 1 × 10^5^ cells were seeded onto the pre-treated glass dishes. Cells were incubated with PE-conjugated rat anti-PD-1 primary antibody (1:200; clone 29 F.1A12; BioLegend) in staining buffer for 30 min on ice. After washing in staining buffer, secondary staining was done with AF647-coupled anti-rat IgG (ThermoFisher, Darmstadt, Germany) antibody for 30 min on ice. Subsequently, cells were washed in staining buffer containing Hoechst 33342 solution (1:5000; ThermoFisher, Darmstadt, Germany), and visualized with a Leica SP5 II confocal microscope (Leica, Wetzlar, Germany).

### Data availability

All data generated or analysed during this study are included in this published article and its supplementary information files.

## Electronic supplementary material

Supplementary Information
