# Supplementary material for: Dying cells expose a nuclear antigen cross-reacting with anti-PD-1 monoclonal antibodies
Source: Sci Rep. 2018 Jun 11;8:8810. doi: 10.1038/s41598-018-27125-6 (PMC5995819; doi:10.1038/s41598-018-27125-6)
Supplement: Supplementary file 1 — Supplementary Information [file 41598_2018_27125_MOESM1_ESM.pdf]

Dying cells expose a nuclear antigen cross-reacting with anti-PD-1 monoclonal antibodies

Philipp Metzger, Sabrina V. Kirchleitner, Lars König, Christine Hörth, Sebastian Kobold, Stefan Endres, Max Schnurr and Peter Duewell

Supplementary information

Supplementary Figure S1: Anti-PD-1 staining of B16-F10 is restricted to cells staining positive for dead cell markers.

a

```
sgRNA2      5´-------------------------------GTTCAGCATAAGATCCTCCG-----------3´
Wild-type   5´-AGTCTGGTTGCTGGGACTCAGGCGGTTCCAGTTCAGCATAAGATCCTCCGACCAGTTGGA-3´
Allele 1    5´-AGTCTGGTTGCTGGGACTCAG--------------GCATAAGATCCTCCGACCAGTTGGA-3´
Allele 1    5´-AGTCTGGTTGCTGGGACTCAG--------------GCATAAGATCCTCCGACCAGTTGGA-3´
Allele 1    5´-AGTCTGGTTGCTGGGACTCAG--------------GCATAAGATCCTCCGACCAGTTGGA-3´
Allele 2    5´-AGTCTGGTTGCTGGGACTCAGGCGGTTCCAG-----CATAAGATCCTCCGACCAGTTGGA-3´
Allele 2    5´-AGTCTGGTTGCTGGGACTCAGGCGGTTCCAG-----CATAAGATCCTCCGACCAGTTGGA-3´
Allele 2    5´-AGTCTGGTTGCTGGGACTCAGGCGGTTCCAG-----CATAAGATCCTCCGACCAGTTGGA-3´
```

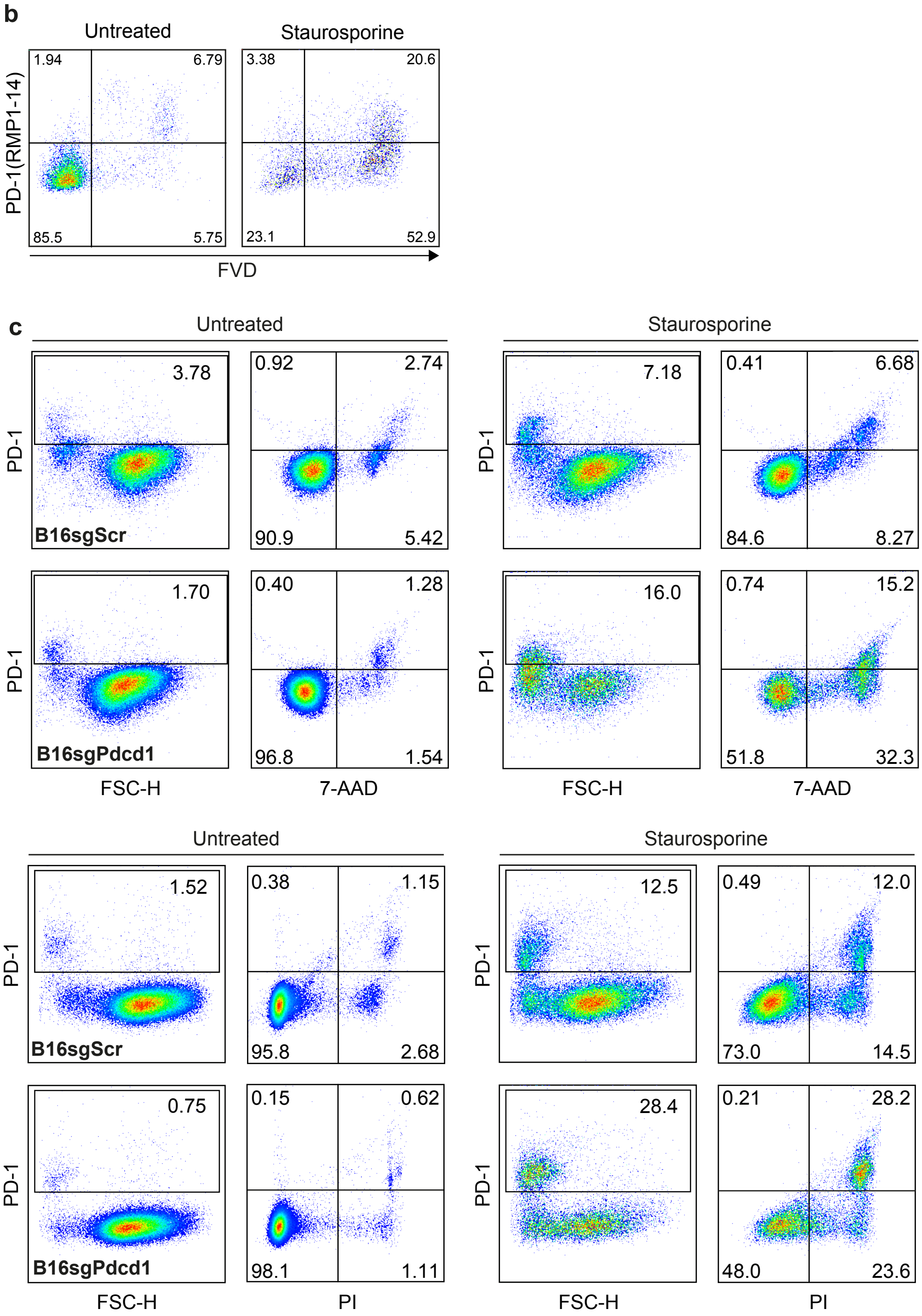

(**a**) Validation of single cell clones by Sanger sequencing of individual alleles. (**b**) B16-F10 cells were cultured in the absence or presence of staurosporine (1 µM). Anti-PD-1 antibody (clone RMP1-14) and fixable viability dye (FVD) staining was analysed by flow cytometry. (**c**) CRISPR/Cas9-targeted B16-F10 control (B16sgScr) and PD-1$^{-/-}$ (B16sgPdcd1) cells were cultured in the absence or presence of staurosporine (1 µM). Anti-PD-1 antibody (clone 29F.1A12) and 7-AAD (upper panel) or propidium iodide (lower panel) was analysed by flow cytometry. Representative data of two independent experiments are depicted.

Supplementary Figure S2: Staining with anti-PD-1 antibodies in various tumour cell lines is restricted to dead cells.

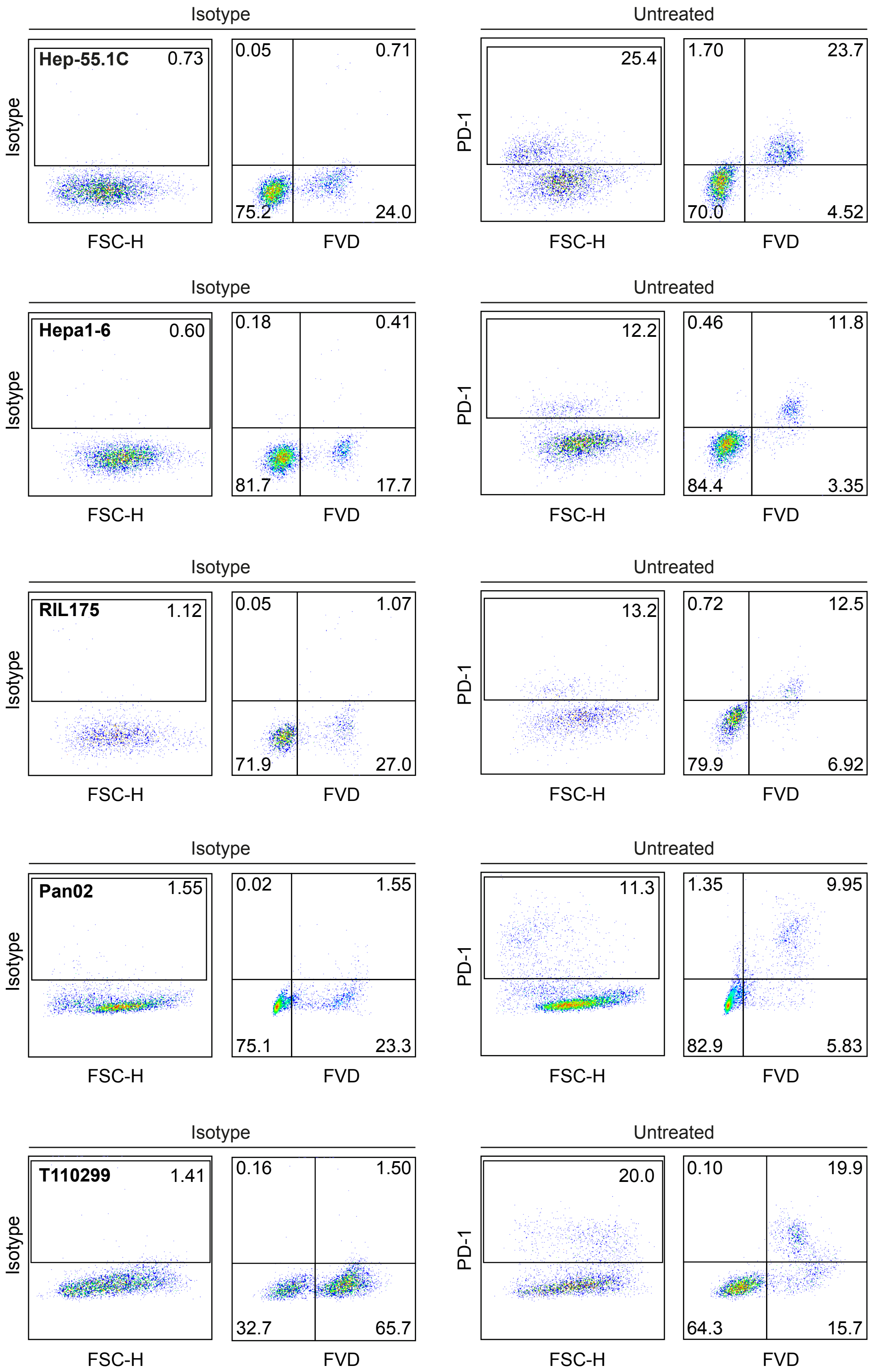

Hepatocellular carcinoma cells (HEP-55.1C; Hepa1-6; RIL176) and pancreatic carcinoma cells (Pan02; T110299) were stained with anti-PD-1 antibody (clone 29F.1A12) and fixable viability dye (FVD). Cells were analysed by flow cytometry. Representative data of one to three independent experiments are depicted.

Supplementary Figure S3: Three-dimensional culture conditions and in vivo growth do not induce PD-1 expression in B16-F10 melanoma cells.

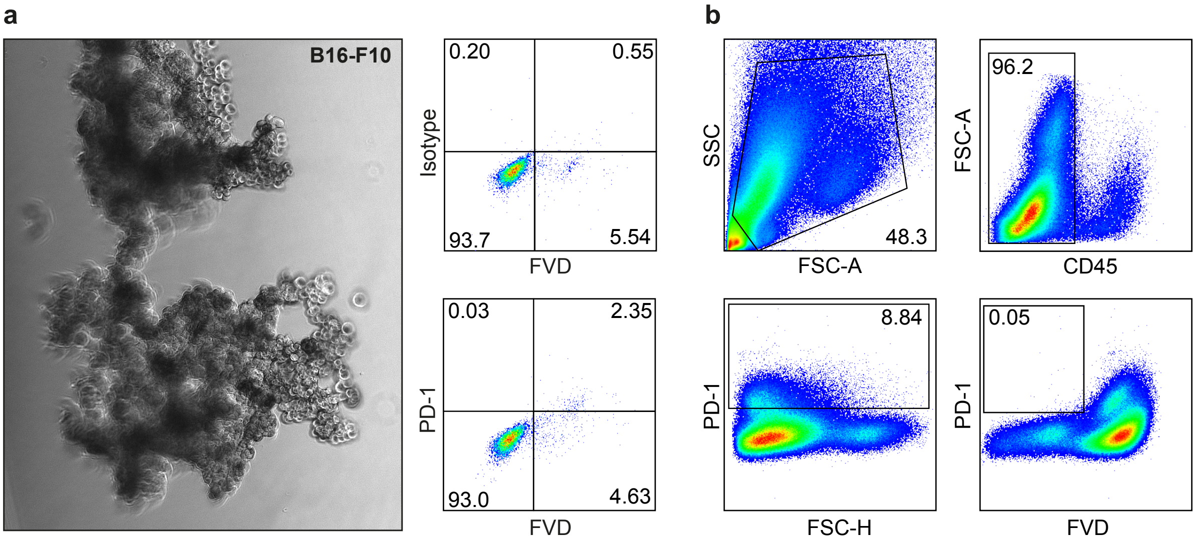

(**a**) B16-F10 single cell suspension was seeded as ´hanging drop` 3D culture for 10 days. PD-1 (clone 29F.1A12) and fixable viability dye (FVD) staining was analysed by flow cytometry. (**b**) In vivo-growing B16-F10 tumours were removed, processed into single cell suspensions and stained with anti-CD45, anti-PD-1 mAb (clone 29F.1A12) and fixable viability dye (FVD). $CD45^{neg}$ cell populations were analysed by flow cytometry.

Supplementary Figure S4: Full-length agarose gel showing qRT-PCR products of cropped blot in Figure-3a.

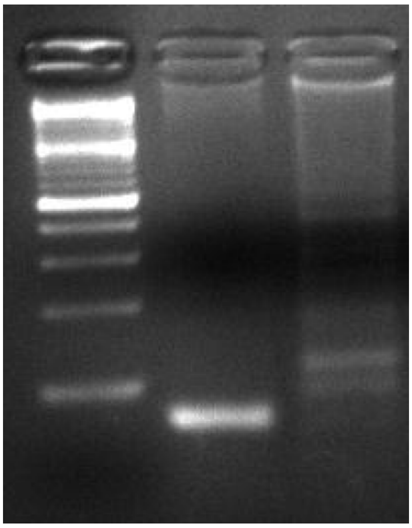

◄ PD-1

Ladder B3Z B16-F10

qRT-PCR reactions of B3Z and B16-F10 cDNA samples were loaded onto a 3 % tris-acetate-EDTA agarose gel and stained with SERVA DNA Stain Clear G. TriDye™ 2-Log DNA Ladder (0.1 - 10.0 kb) shows expected Pdcd1 band size at ~ 77 bp.
